# Tofacitinib Suppresses Natural Killer Cells *In Vitro* and *In Vivo*: Implications for Amyotrophic Lateral Sclerosis

**DOI:** 10.3389/fimmu.2022.773288

**Published:** 2022-02-07

**Authors:** Claudia Figueroa-Romero, Alina Monteagudo, Benjamin J. Murdock, Joshua P. Famie, Ian F. Webber-Davis, Caroline E. Piecuch, Samuel J. Teener, Crystal Pacut, Stephen A. Goutman, Eva L. Feldman

**Affiliations:** Department of Neurology, University of Michigan, Ann Arbor, MI, United States

**Keywords:** ALS, NK cells, immune system, tofacitinib, JAK/STAT

## Abstract

Amyotrophic lateral sclerosis (ALS) is a fatal and incurable neurodegenerative disease with few therapeutic options. However, the immune system, including natural killer (NK) cells, is linked to ALS progression and may constitute a viable therapeutic ALS target. Tofacitinib is an FDA-approved immunomodulating small molecule which suppresses immune cell function by blocking proinflammatory cytokine signaling. This includes the cytokine IL-15 which is the primary cytokine associated with NK cell function and proliferation. However, the impact of tofacitinib on NK activation and cytotoxicity has not been thoroughly investigated, particularly in ALS. We therefore tested the ability of tofacitinib to suppress cytotoxicity and cytokine production in an NK cell line and in primary NK cells derived from control and ALS participants. We also investigated whether tofacitinib protected ALS neurons from NK cell cytotoxicity. Finally, we conducted a comprehensive pharmacokinetic study of tofacitinib in mice and tested the feasibility of administration formulated in chow. Success was assessed through the impact of tofacitinib on peripheral NK cell levels in mice. We found tofacitinib suppressed IL-15-induced activation as measured by STAT1 phosphorylation, cytotoxicity, pro-inflammatory gene expression, and pro-inflammatory cytokine secretion in both an NK cell line and primary NK cells. Furthermore, tofacitinib protected ALS neurons from NK cell-mediated cytotoxicity. In mice, we found tofacitinib bioavailability was 37% in both male and female mice; using these data we formulated mouse containing low and high doses of tofacitinib and found that the drug suppressed peripheral NK cell levels in a dose-dependent manner. These results demonstrate that tofacitinib can suppress NK cell function and may be a viable therapeutic strategy for ALS.

## Introduction

Amyotrophic lateral sclerosis (ALS) is a progressive neurodegenerative disease resulting in death of the motor neurons (1). The average patient lifespan is 2 to 4 years from disease diagnosis, and few therapeutic options exist. However, an increasing body of literature suggests that the immune system is involved in the pathogenesis of ALS (2, 3), with specific immune cell populations likely contributing to disease progression in ALS mouse models (4–9). Similarly, in human ALS patients, changes in peripheral immune cell numbers and activation state correlate with disease progression (10–13). Unfortunately, suppressing the immune system can have unintended and sometimes fatal consequences. General immune suppression increases susceptibility to pathogens and cancer (14); it may also accelerate ALS progression (15, 16) since several immune populations perform protective functions, which slow disease progression (10, 11). The loss of protective immune cell populations likely explains previous failures of immunosuppressive drugs for ALS. Conversely, certain immune cell populations accelerate ALS (6–10, 12, 13); thus, targeting these specific immune populations may be a more nuanced and potentially effective approach for slowing disease progression than global immune suppression.

Generally, immune cells do not attack the body’s own cells under homeostatic conditions. However, natural killer (NK) cells destroy the body’s own cells when they become cancerous, infected, or damaged (17, 18). NK cells may also contribute to ALS progression (7, 8, 10, 13); we and others have found elevated NK cell levels in the peripheral blood of ALS patients (10, 11) and NK cells accumulate in the spinal cord of ALS mice (7, 8, 19). Moreover, during ALS, motor neurons stop expressing major histocompatibility complex proteins, which mark them as self, protecting them from NK cell-mediated cytotoxicity (20, 21). This suggests a particular vulnerability of motor neurons to NK cells in ALS. Finally, NK cells drive a pro-inflammatory microglia phenotype and simultaneously suppress protective regulatory T cells during ALS (7). Thus, drugs targeting NK cells may prove a viable therapeutic option for ALS, both by blocking NK cell cytotoxicity as well as preventing a pro-inflammatory cascade in the central nervous system.

Tofacitinib is a small molecule pharmaceutical approved for treating multiple immune disorders, including rheumatoid arthritis (22), ulcerative colitis (23), and psoriasis (24). The drug suppresses pro-inflammatory immune activation by blocking the JAK/STAT pathway of the adaptive immune system (25, 26) while preserving innate immune activity and regulatory function (27, 28). However, cytokines associated with NK cell survival and function, including IL-15, signal through the JAK/STAT pathway as well (29–32) and would also be blocked by tofacitinib (33). Indeed, several studies suggest tofacitinib suppresses NK cell numbers in the peripheral blood of mice (34, 35) and humans (36). However, little research has been performed to examine the impact of tofacitinib on NK cell activation. Thus, tofacitinib could potentially block NK cell cytokine production and cytotoxicity in addition to lowering overall levels, providing added benefits as an ALS treatment. In addition, tofacitinib would target NK cells and pro-inflammatory pathways while preserving protective immune function (28) thus overcoming previous failures of previous immune-based therapies for ALS (37).

The present study therefore evaluated the ability of tofacitinib to suppress NK cell cytokine expression and cytotoxicity *in vitro*, both in an NK cell line and in primary NK cells derived from ALS participants. We also investigated whether tofacitinib suppressed NK cell cytotoxicity to inducible neurons (iNeurons) differentiated from ALS patient-derived inducible pluripotent stem cells (iPSCs). Finally, we examined tofacitinib pharmacokinetics in mice as well as the impact *in vivo* on the immune system, since these data have not been previously established and are crucial to future preclinical studies of ALS. Our study found that tofacitinib suppresses NK cell cytotoxicity and cytokine production *in vitro* and suppresses NK cell levels *in vivo* in mice after oral administration in food. These data demonstrate that tofacitinib may be a viable ALS treatment and establish a foundation for future preclinical studies.

## Methods

### Study Participants

Healthy control participants without a history of neurodegenerative disease, chronic inflammatory disease, collagen vascular disease, or immunomodulatory medication use were recruited through the University of Michigan Institute for Clinical & Health Research. In parallel, ALS participants meeting a diagnosis of ALS by El Escorial Criteria were recruited during clinical visits at the University of Michigan Pranger ALS Clinic as previously described (13). All study participants provided oral and written informed consent and the study received ethics board approval by the University of Michigan Medical School Institutional Review Board (HUM00028826).

### Cell Lines and Primary Human NK Cells

#### Cell Lines

The NK-92 NK cell line (ATCC Cat# CRL-2408, RRID : CVCL_3755) and K-562 leukemia cell line (ATCC Cat# CCL-243, RRID : CVCL_0004) were acquired from ATCC (Manassas, VA). NK-92 cells were grown in NK media [Alpha’s Modification of Medium Essential Eagle media (STEMCELL Technologies cat #36453) supplemented with 12.5% horse serum (Gibco cat #16050122), 12.50% fetal bovine serum (FBS, Sigma Aldrich cat #F4135), 1% penicillin/streptomycin (Gibco cat #15140122), 0.2 mM myo-inositol (Sigma-Aldrich cat #17508), 0.02 mM folic acid (Sigma-Aldrich cat #F8758), and 0.1 mM β-mercaptoethanol (Sigma cat #M7522) and 645.2 nM IL-2 (PeproTech cat #200-02)]. K-562 cancer cells were grown in K-562 media [Iscove’s Modified Dulbecco’s Medium (STEMCELL Technologies cat #36150) with 10% FBS and 1% penicillin/streptomycin (Gibco cat #15140122)]. Human-derived iPSC lines #1021 (control) and #265 (sporadic ALS, sALS) were obtained from the University of Michigan ALS Biorepository (38). Control and ALS iPSCs were used to generate iNeurons by suppressing the polypyrimidine-tract-binding (PTB) protein, as previously described (39). Briefly, iPSCs were cultured on poly-D-lysine (50 µg/L, Sigma cat #p1149)/laminin (1:100, Sigma cat #L2020) coated plates in iPSC media [E8 media (Gibco cat #A1517001) supplemented with iROCK Y27632 (Fisher cat #BDB562822)] in 6-well plates at a density of 1x10^5^ cells/well. The following day (Day 1), the media was changed to iNeuron media #1 [E8 media supplemented with 1X N2 supplement (Gibco, cat #17502-048), 1X NEAA supplement (Gibco cat #11140-050), 10 ng/mL BDNF (Peprotech cat #450-02), 10 ng/mL NT3 (Peprotech cat #450-03), 0.2 µg/mL mouse laminin (Sigma cat #L2020), 2 mg/mL doxycycline (Sigma cat #D3447)]. On Day 2, the cells were changed to iNeuron media #2 [½ E8, ½ DMEM/F12 (Gibco cat #11320-033), 1X N2 Supplement, 1X NEAA supplement, 10 ng/mL BDNF, 10 ng/mL NT3, 0.2 µg/mL laminin, 2 mg/mL doxycycline]. On Day 3, cells were changed to iNeuron media #3 [Neurobasal-A (Gibco cat #12349-015), 1X B27 supplement (Gibco cat #17504-044), 1X Glutamax supplement (Gibco cat #35050-061), 10 ng/mL BDNF, 10 ng/mL NT3, 0.2 µg/mL mouse laminin, 2 mg/mL doxycycline]. Additional media #3 was added on Day 6 and Day 8. iNeurons were differentiated for 10 days prior to treatment.

#### Primary NK Cells

10 mL of whole blood was collected from control and ALS participants, as previously described (10, 12, 13). NK cells were enriched using RosetteSep Human NK isolation cocktail (STEMCELL Technologies cat #15025) and cultured in NK media supplemented with 645.2 nM IL-2 or IL-2 + 2.33 nM IL-15 (PeproTech cat #200-15) for co-culture assays. All cells were grown at 37°C in 5% CO_2_.

### NK-92 IL-15 Stimulation and Tofacitinib Treatment Paradigms

**Figure 1:**
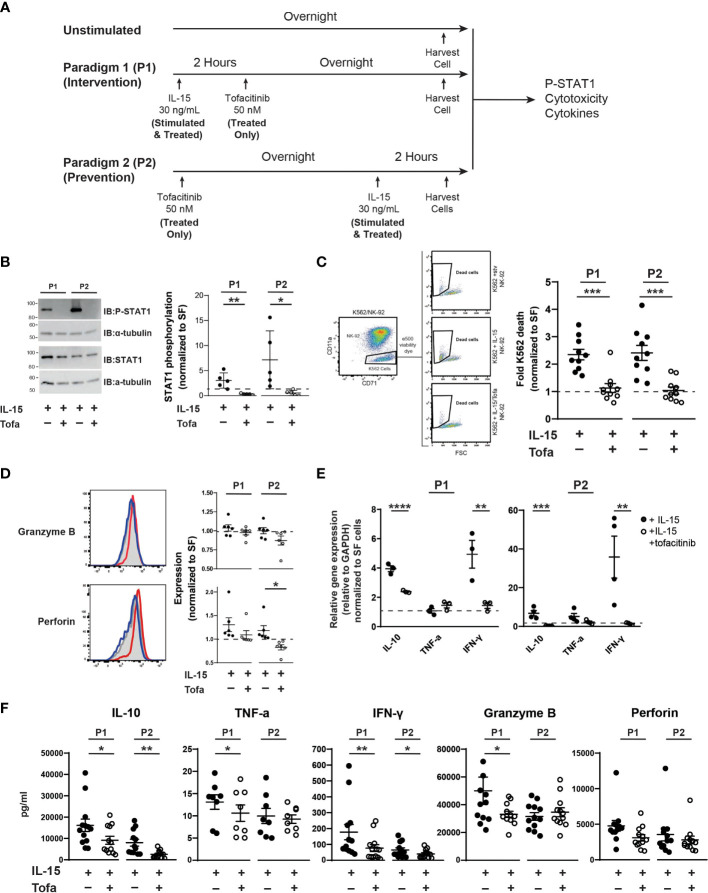
Tofacitinib inhibits NK-92 cells function *in vitro*. **(A)** NK-92 cells were cultured with serum-free media under two culture paradigms: Paradigm 1 (P1) intervention treatment, whereby NK-92 cells were treated with IL-15 for two hours and then cultured overnight in the presence of tofacitinib (Treated), and Paradigm 2 (P2) prevention treatment where NK-92 cells were treated overnight with tofacitinib prior to two-hour IL-15 stimulation. **(B)** Stimulated and Treated NK-92 cells were assessed for STAT1 phosphorylation (P-STAT) following both culture paradigms. Representative immunoblots for Stimulated and Treated P-STAT1 and α-tubulin (internal reference) are shown. Quantitative data represent densitometric analysis, where total STAT1 and P-STAT1 signals were first normalized to α-tubulin then to P-STAT1 levels in Unstimulated cells; n = 5 independent experiments. **(C)** Unstimulated, Stimulated, and Treated NK-92 cells were co-cultured with K-562 cancer cells (10:1 ratio) following initial P1 or P2 culture paradigms. Flow cytometry was used to identify K-562 cells in the co-culture, and cell death was assessed by viability dye staining. K-562 cell death was quantitated for Stimulated and Treated NK-92 cells and normalized to Unstimulated NK-92 cells; n = 10 independent experiments. **(D)** Expression of the intracellular NK cell proteins perforin and granzyme B was determined by flow cytometry on Unstimulated, Stimulated, and Treated NK-92 cells using intracellular flow cytometry following P1 and P2 culture paradigms. Representative histograms are shown; gray peaks = Unstimulated NK-92 cells, red peaks = Stimulated NK-92 cells, blue peaks = Treated NK-92 cells show MFI. MFI from Stimulated and Treated cells were normalized to protein levels in Unstimulated NK-92 cells for quantitation; n = 6 independent experiments. **(E)** Gene expression of IL-10, TNF-α, and IFNγ cytokines was assessed in Unstimulated, Stimulated, and Treated NK-92 cells following P1 and P2 paradigms using qRT-PCR. Data for Stimulated and Treated cells were normalized to GAPDH expression then to the Unstimulated cells; n = 3-4 independent experiments. **(F)** Extracellular expression of IL-10, TNF-α, IFN-γ, granzyme B, and perforin was assessed for Stimulated and Treated NK-92 cells using a CD8/NK cell multiplex analysis following P1 and P2 treatment paradigms (n = 8-13 independent experiments). For all experiments, quantitative data is shown as the mean ± SEM; **(A–E)** comparisons were made by Student’s t-test; **(F)** comparisons were made using a paired t-test or a Wilcoxon test based on normality of the data. Horizontal dashed lines represent normalized Unstimulated NK-92 cell levels. *P<0.05, **P<0.01, ***P<0.001, ****P<0.0001.

NK-92 cells were cultured using two IL-15/tofacitinib paradigms (**Figure 1A**). In the first paradigm (P1), NK-92 cells were cultured for two hours with 2.33 nM IL-15 in serum-free NK media prior to overnight treatment with 50 nM tofacitinib (Selleckchem cat #CP-690550). In the second treatment paradigm (P2), NK-92 cells were cultured overnight with 50 nM tofacitinib in serum-free NK media prior to two-hour culture with 2.33 nM IL-15. 50 nM concentration of tofacitinib was used based on previous *in vitro* immune studies (40, 41). For each treatment paradigm, three groups of NK-92 cells were generated: cells receiving no IL-15 and no tofacitinib (Unstimulated), cells receiving only IL-15 (Stimulated) or cells receiving IL-15 stimulation and tofacitinib treatment (Treated). NK-92 cells were then collected, washed, and analyzed for STAT1 phosphorylation (P-STAT), cytotoxicity towards K-562 cells, granzyme B and perforin expression, or cytokine gene expression (see below).

### Primary NK Cell Stimulation and Tofacitinib Treatment

1x10^5^ human primary NK cells were cultured in a 48-well plate (Corning cat #3524) in NK media supplemented with 645.2 nM IL-2 + 2.33 nM IL-15 ± 50 nM tofacitinib for two hours at 37°C in 10% CO_2_. Unlike NK-92 cells, primary NK cells were provided additional IL-2 cytokine stimulation to enhance survival in culture (data not shown) (42). The cells were washed and pelleted for subsequent western blot, quantitative real-time PCR (qRT-PCR), and cytotoxicity analysis.

### Western Blot Analysis

NK-92 or primary NK cells were lysed in RIPA buffer (Pierce/Thermo Fisher Scientific cat #89901) supplemented with cOmplete mini, EDTA-free protease inhibitors (Roche cat #11836170001), sonicated, and centrifuged. Protein samples were resolved by SDS-PAGE in 10% acrylamide gels, transferred to Immobilon-FL PDVF membranes (Millipore cat #IPFL00010), and immunoblotted with the indicated primary antibodies: rabbit anti-STAT1 (D1K94) (Cell Signaling Technologies, CST, cat #14994S), rabbit anti-P-STAT1 (Y701) (CST cat #9167S), and rat anti-α-tubulin (Abcam cat #ab6160; RRID : AB_305328). Goat anti-rabbit (cat #7074S; RRID : AB_2099233) and anti-biotin horseradish peroxidase (HRP)-linked (cat #7075P5) secondary antibodies were used at 1:2000 (CST) and anti-rat IgG HRP-conjugated secondary antibody was used at 1:5000 (R&D cat #HAF005; RRID : AB_1512258). Densitometric analysis was performed Quantity One v.4.6.5 (Bio-Rad).

### Cytotoxicity Assays

#### NK-92 Cells and K-562 Cells

Pre-treated NK-92 cells (Paradigm 1 or 2) were plated at a density of 1x10^6^ NK-92 cells and co-cultured with 1x10^5^ K-562 cells for two hours at a 10:1 ratio in a final volume of 500 μL followed by flow cytometric analysis of K-562 and NK-92 cell viability dye levels (positive levels indicating cell death, see below).

#### NK-92 Cells and iNeurons

1x10^6^ NK-92 cells were starved for 2 hours in serum-free NK media, then treated with 50 nM tofacitinib or vehicle (dimethyl sulfoxide, DMSO) for 30 minutes prior to stimulation with 2.33 nM IL-15 for 4 hours (similar to the intervention treatment paradigm, P2). Treated NK-92 cells were re-suspended in 0.5 mL iNeuron media #3 and co-cultured for 2 hours with 10-day old iNeurons plated at 2x10^5^ cells/well (NK-92:iNeuron = 5:1). At the end of the incubation, the media containing the NK-92 was removed and the iNeurons were washed with 1X PBS and released from the plates with Accutase (Innovative Cell Technologies cat #AT-104) for viability analysis *via* flow cytometry (see below).

#### Primary NK Cells and K-562 Cells

1x10^5^ human primary NK cells were co-cultured with 1x10^5^ K-562 cells at a 1:1 ratio were seeded to a 48-well plate (Corning) with NK media for 2 hours at 37°C in 5% CO_2_ under one of three conditions: with 645.2 nM IL-2, with IL-2 + 2.33 nM IL-15, or with IL2 + IL-15 + 50 nM tofacitinib. Following co-culture, conditioned media was then collected and stored for subsequent analysis (see below). K-562 and primary NK cells were then washed with flow buffer [1000 mL 1X PBS + 20 mL FBS + 0.01 g NaN_3_] and plated for analysis of both cell types using flow cytometry and viability dye (see below).

### Quantitative Real-Time PCR (qRT-PCR)

RNA was isolated from NK-92 or primary NK cells using the RNeasy isolation kit (Qiagen cat #74104) and with RNA/DNA/RNase-free DNase treatment (Qiagen cat#79254). cDNA was generated using 0.5 μg of NK-92 RNA or 40 ng primary NK cell RNA and 5X iScript RT (Bio-Rad cat#1708840) in a 20 μL reaction following the manufacturer’s protocol. The reactions were run in a PTC-200 Peltier Thermal Cycler (MJ Research). qRT-PCR was performed in triplicate using 10-μL reactions consisting of sequence specific TaqMan™ primers for human IL-10 (Hs00961622_m1), TNF-α (Hs00174128), IFN-γ (Hs00989291_m1), GAPDH (Hs02758991_g1), and yWHAZ (Hs03044281_g1); 2X gene expression Master Mix (Applied Biosystems/Thermo Fisher Scientific, cat #4369016) and 2 μL cDNA. C_T_ values were used to calculate ΔC_T_ and ΔΔC_T_ using GAPDH or yWHAZ as the internal references. Data were expressed as the mean of the relative quantity of gene expression (2^−ΔΔCT^).

### Multiplex Analysis of Cytokines and Secreted Proteins

The release of cytokines and pro-apoptotic factors by NK-92 and primary NK cells was assessed using the LEGENDplex Human CD8/NK Cell Panel (Biolegend, cat# 740267) according to the manufacturer’s instructions. In brief, fluorescent beads were incubated with conditioned media from NK-92 or primary NK cells co-cultured with K-562 cancer cells, and flow cytometry (see below) was used to quantify IL-10, TNF-α, IFN-γ, granzyme B, and perforin in the conditioned media.

### Mice

Mice were purchased from Jackson Laboratory (Bar Harbor, ME). Male and female C57BL/6 mice (Stock #000664; RRID : IMSR_JAX:000664) were used for initial tofacitinib pharmacokinetic assays. For tofacitinib efficacy studies, male and female non-carrier, wild-type (WT) control littermates of SOD1^G93A^ ALS mice were used (B6.Cg-Tg(SOD1*G93A)1Gur/J; Jackson Stock #004435). All mice were housed under specific pathogen-free conditions. Animals were fed 5L0D chow *ad libitum* when not treated. All mouse studies were performed in accordance with University of Michigan Institutional Animal Care & Use Committee approved protocols (approval #PRO00010247). Mouse studies were conducted in accordance with the United States Public Health Service’s policy on Humane Care and Use of Laboratory Animals.

### Tofacitinib Administration to Mice and Plasma Collection for Pharmacokinetic Assays

Tofacitinib was suspended at 2 mg/mL in PBS containing 5% DMSO and 10% PEG-400, which was administered by intravenous (IV) injection (10 mg/kg, 10 mice) or *per os* (PO) *via* gavage (20 mg/kg, 10 mice). At the given time points (0.083, 0.167, 0.25, 0.5, 1, 2, 4, 7, 16, and 24 hours), blood samples were collected using heparinized calibrated pipettes. Samples were centrifuged at 2000*g* for 10 minutes. Subsequently, plasma was collected from the upper layer and frozen at -80°C for later analysis.

### Liquid Chromatography

#### Sample Preparation

To precipitate plasma proteins, 150 μL of acetonitrile containing internal standard and 30 μL of ice-cold acetonitrile were added to 30 μL of plasma. The mixture was vortexed for 10 minutes and centrifuged at 15,000 x g for 10 minutes. The supernatant was transferred to a 96-well plate (Fisher Scientific) for liquid chromatograph-tandem mass spectrometry (LC–MS/MS).

#### Sample Specificity

The chromatograms of blank plasma versus blank plasma spiked with internal standard (CE302) showed that the blank plasma did not interfere with tofacitinib and internal standard determination.

#### Calibration Curve

Analytical curves were constructed with 12 nonzero standards by plotting the tofacitinib peak area ratio to the internal standard versus the concentration in plasma. The concentration range was evaluated from 1 to 10000 ng/mL for drug level quantification in plasma. A blank sample (matrix sample processed without internal standard) was used to exclude contamination or interference. The curve was built with linear regression with weighing (1/X^2^). The linearity of the relationship between peak area ratio and concentration was demonstrated by the correlation coefficients (r = 0.9990).

#### Quality Control (QC) Samples

The accuracy and precision were evaluated at four concentration levels (2 ng/mL, 400 ng/mL, 4500 ng/mL, and 9000 ng/mL) with three individual replicates at each concentration. The QC stock solution was prepared from separate weighing. QC samples were prepared at four levels (2 ng/mL, 400 ng/mL, 4500 ng/mL, and 9000 ng/mL). QC samples were run before, in the middle, and after running the samples. At least 50% of QCs at each level were within 15% of their nominal concentration. The intra-batch precision was calculated and expressed as relative standard deviation. Data indicate that the assay method was reliable and reproducible.

#### Analysis

Tofacitinib concentrations in mouse plasma were determined by a liquid chromatography tandem mass spectrometry (LC–MS/MS) method developed and validated for this study. The LC-MS/MS method was preformed using an AB-4500 Qtrap (Sciex, Concord, ON, Canada) mass spectrometer with electrospray ionization source interfaced with a Shimadzu high-performance LC system. Separation was performed on an XBridge C18 column (50 × 2.1 mm ID, 3.5 µm; Waters, Milford, MA, USA) at a flow rate of 0.4 mL/minute. The mobile phase consisted of A (water with 0.1% formic acid) and B (acetonitrile with 0.1% formic acid). The gradient was 0.0-0.5 minutes, 2% B; 0.5-2.0 minutes, 2-95% B; 2.0-3.6 minutes, 95% B; and 3.6-4.1 minutes, 95-2% B. The mass spectrometer was operated in positive mode with multiple reaction monitoring for analysis. The multiple reaction monitoring transitions were m/z 313.1 > 173.1 for tofacitinib and 455.2 > 425.2 for the internal standard. The gas temperature was 500°C with an ionspray voltage of 5500 V, gas 1 and gas 2 of 30 psi, and curtain gas of 30 psi. Analyst Software (version 1.6) from Applied Biosystems (MDS SCIEX; Carlsbad, CA, USA) was used to control the LC-MS/MS system, as well as for data acquisition and processing. All pharmacokinetic parameters were estimated using non-compartmental analyses with Phoenix WinNonlin software (Certara, Princeton, NJ).

### Tofacitinib Mouse Chow

Low-dose (5 mg/kg) and high-dose (30 mg/kg) chow was manufactured by Research Diets (New Brunswick, NJ). To determine the initial tofacitinib content in the chow, chow from two cages of mice was weighed daily for a week to determine the daily chow consumption per cage. The average chow consumption for each mouse was then calculated, and low- and high-dose chow was formulated based on average consumption. For the peripheral immune analysis, male and female mice were placed on low-dose, high-dose, or normal chow (control animals) for two weeks prior to harvest.

### Blood Leukocyte Collection

Peripheral immune cells were harvested as previously described (9). At the time of harvest, mice were euthanized with sodium pentobarbital, whole blood was collected from the vena cava and measured using a 1 mL syringe (BD Biosciences, Franklin Lakes, NJ), and transferred to a BD Vacutainer^®^ blood collection tube (BD Biosciences) coated with 3.6 mg of EDTA. Red blood cells were lysed with 9.5 mL red blood cell lysis buffer [150 mM NH_4_Cl, 10 mM KHCO_3_, 0.1 mM EDTA (Thermo Fisher Scientific) with 13.8 mM HEPES, pH 7.2-7.5 (Thermo Fisher Scientific)] for 20 minutes on a rocker at room temperature. Leukocytes were pelleted (1000 rpm, 10 minutes, 4°C, with brake), supernatant siphoned off, washed twice with flow cytometry buffer [1X PBS, 2% FBS (Thermo Fisher Scientific), 0.1% NaN_3_] and resuspended in 1 mL flow cytometry buffer. Cells were counted by hemocytometer (Hausser Scientific, Horsham, PA), and kept on ice until staining for flow cytometry.

### Flow Cytometry

#### Intracellular Perforin and Granzyme B Staining of NK-92 Cells

All samples were washed and resuspended at a density of ≤10^6^ cells/25 µL, plated in U-bottom 96-well plates (Fischer cat #07-200-760), and spun down at 1200 rpm for 10 minutes. Samples were incubated with 10 µg/mL human TruStain FcX™ blocking solution (Biolegend cat #422302; RRID : AB_2818986) at 4°C for 30 minutes prior to immune staining. Following blocking stage, NK-92 cells were stained with CD56 and HLA and washed with flow buffer (PBS + 2% FBS). Cells were then permeabilized using Cytofix/cytoperm (BD cat #554714; RRID : AB_2869008) and stained with antibody for perforin (Biolegend cat #353303; RRID : AB_10915476) and granzyme B (Biolegend cat #515403; RRID : AB_2114575). Control stains were performed with non-specific IgG antibody (Biolegend cat #400111; RRID : AB_2847829 and #400137). Cells were then transferred to polystyrene tubes (12x75 mm) (BD Biosciences) and analyzed on a BD LSRFortessa™ flow cytometer with FACSDiva™ software (BD Biosciences) and FlowJo (FlowJo, Ashland, OR).

#### NK-92 and Primary NK Cytotoxicity Assays

Following collection and wash, co-cultures of NK and K-562 cells were incubated with Fixable Viability Dye eFluor™ 506 (1:500) (eBioscience cat #65-0866-14) during the blocking stage. Samples were then incubated with surface stains CD56 (Biolegend cat #318318; RRID : AB_604107), CD11a (Biolegend cat#301207; RRID : AB_10660819), and CD71 (Biolegend cat #334110; RRID : AB_2563117) at 4°C for 30 minutes. K-562 cells were identified as CD56-, CD11a-, CD71+. Samples were fixed using Stabilizing Fixative (BD cat #338036; 1:3 dilution).

#### iNeurons

All samples were washed, resuspended at a density of ≤10^6^ cells/25 µL, plated in U-bottom 96-well plates, and blocked at 4°C for 30 minutes prior to staining. Cells were then stained for annexin V (Invitrogen cat #12-8102-69) and with the viability dye 7-amino-actinomycin D (7AAD; Invitrogen cat #00-6993-50), according to the manufacturer’s instructions, and fixed for analysis, as above, consistent with previously reported protocols in neurons (43, 44).

#### Mouse Peripheral Immune Cells

All samples were washed and resuspended at a density of ≤10^6^ cells/25 µL, plated in U-bottom 96-well plates (Fischer), and spun down at 1200 rpm for 10 minutes. Samples were incubated with 10 µg/mL mouse TruStain FcX™ blocking solution (Biolegend cat #101320; RRID : AB_1574975) at 4°C for 30 minutes prior to staining. Samples were incubated with antibodies against myeloid and lymphoid surface markers as previously described (9). In brief, cells were stained with antibodies against CD45, CD11b, Ly6C, and Ly6G to analyze myeloid populations and CD45, CD3, CD4, CD8, NK1.1, and CD49b to analyze lymphocyte and NK cell populations.

### Statistics

All statistics were performed using GraphPad Prism version 8.0.0 (San Diego, CA). All datasets were assessed for normality using normality using Shapiro-Wilk (45). For comparing between two groups, either a two-tailed Student’s t-test (normally distributed data) or Mann-Whitney (non-normally distributed data) were used. For cytokine expression by NK-92 cells a paired t-test or a Wilcoxon test (paired data with non-normal distribution) was used when data was not normally distributed. A Wilcoxon test was used for comparing NK-92 cytotoxicity to iNeurons and comparing primary NK cell cytotoxicity to K-562 cancer cells. For comparing peripheral immune cell levels, two-way ANOVA with multiple comparisons was used. *P*-values < 0.05 were considered statistically significant.

## Results

### Tofacitinib Inhibits NK-92 Cells Function *In Vitro*

Previous studies demonstrate that tofacitinib lowers NK cell levels *in vivo* (34–36, 46); however, little is known about the effect of tofacitinib on NK cell activation. Blocking NK cell activation in addition to lowering levels could potentially increase tofacitinib efficacy for treating NK cell-mediated diseases, such as ALS. Thus, we explored whether tofacitinib reduces NK cell cytotoxicity, cytokine production, and trafficking *in vitro*. We initially employed a commercially available NK cell line, NK-92 cells, and two treatment paradigms to explore the impact of tofacitinib on NK cell function (**Figure 1A**). In the first treatment paradigm (P1, intervention treatment) NK-92 cells were activated with IL-15 for two hours (30–32) and cultured overnight in the presence of tofacitinib. In the second paradigm (P2, prevention treatment), NK-92 cells were first pre-treated with tofacitinib overnight and then activated for two hours with IL-15. In parallel, Unstimulated cells (which received no IL-15 stimulation, serum stimulation, or tofacitinib treatment) were also cultured overnight and analyzed simultaneously with P1 or P2 NK-92 cells. For both paradigms, NK-92 cells were further divided into two groups: NK-92 cells cultured with IL-15 without tofacitinib (Stimulated) and NK-92 cells cultured with both IL-15 and tofacitinib (Treated). This resulted in a total of five NK-92 groups (Unstimulated, P1 Stimulated, P1 Treated, P2 Stimulated, and P2 treated) that were analyzed in parallel.

First, we confirmed that tofacitinib treatment blocks IL-15 signaling in NK-92 cells in both intervention and prevention paradigms. Tofacitinib disrupts JAK/STAT signaling by blocking STAT protein phosphorylation (47). Therefore, we measured phosphorylated STAT1 (P-STAT1) levels in Unstimulated, Stimulated, and Treated NK cells and normalized values levels in Untreated NK-92 cells to account for run-to-run variation. Immunoblot analysis indicated that tofacitinib treatment significantly reduced P-STAT1 in both culture paradigms (10-fold in P1 and 13 fold in P2, **Figure 1B**) demonstrating that tofacitinib blocks IL-15 signaling in NK-92 cells.

We next tested whether tofacitinib treatment suppresses NK cell function by assessing cytotoxicity, the intrinsic ability of NK-92 cells to eliminate other cells. NK-92 cells in both intervention and prevention treatment paradigms (Unstimulated, Stimulated, or Treated) were co-cultured with K-562 leukemia cells. K652 cell death, as measured by cellular viability dye *via* flow cytometry, was used to quantify NK-92-killing activity (48) (**Figure 1C**); cytotoxicity of Stimulated and Treated NK-92 cells was normalized to Unstimulated NK-92 cells. We found that IL-15-treated NK-92 cells doubled the rate of K-562 cancer cells killing under both paradigms. However, under both intervention and prevention treatment paradigms, blocking IL-15 with tofacitinib significantly reduced the ability of NK-92 cells to induce K-562 cell death, showing that tofacitinib reduces the ability of NK cells to eliminate target cells. To ensure that tofacitinib is suppressing NK-92 cytotoxicity rather than reducing cellular viability, we also examined NK-92 survival using viability dye. We found no significant differences in NK-92 viability following tofacitinib treatment (data not shown).

To support these findings, we next examined the impact of tofacitinib on NK-92 expression of intracellular granzyme B and perforin, both of which play key roles in NK cell-mediated cytotoxicity (49). Intracellular granzyme B and perforin levels from Unstimulated, Stimulated, or Treated NK-92 cells (**Figure 1D**) were quantified by the median fluorescent intensity (MFI) from intracellular flow cytometry; values from Stimulated and Treated NK-92 cells were then normalized to the MFI from Unstimulated NK-92 cells. We found that tofacitinib significantly lowered intracellular perforin levels in the prevention paradigm (P2), with a trending reduction in the intervention paradigm (P1). Tofacitinib also induced a trending reduction towards reduced granzyme B levels. These data suggest tofacitinib may reduce NK cell cytotoxicity, partly by suppressing the expression of proteins that induce target cell death.

In addition to direct cytotoxicity, we examined the ability of tofacitinib to suppress NK cell cytokine production, which enhances neuroinflammation in ALS (7). NK-92 cells were cultured under both paradigms, and cytokine IL-10, TNF-α, and IFN-γ mRNA expression levels were measured by qRT-PCR and normalized to Unstimulated cells. Overall, *TNF-α, IL-10*, and *IFN-γ* gene expression increased in both paradigms following IL-15 stimulation, but this increase was reversed by tofacitinib treatment (**Figure 1E**). To confirm these findings, we next examined the secretion of pro-inflammatory and pro-apoptotic factors using a bead-based multiplex analysis paired with flow cytometry. Conditioned media from Stimulated and Treated culture conditions was analyzed for levels of IL-10, TNF-α, IFN-γ, granzyme B, and perforin. Similar patterns of NK cell suppression were seen following both tofacitinib treatment paradigms. As observed with qRT-PCR, both IL-10 and IFN-γ levels were suppressed during the P1 and P2 treatment paradigms (**Figure 1F**). Similarly, granzyme B and perforin expression were suppressed following the P2 treatment paradigm, demonstrating that tofacitinib suppresses the release of pro-inflammatory and pro-apoptotic factors by NK-92 cells. Together, these data demonstrate that tofacitinib suppresses the ability of NK cells to generate cytokines in addition to blocking their cytotoxicity in response to pro-inflammatory stimulation.

**Figure 2:**
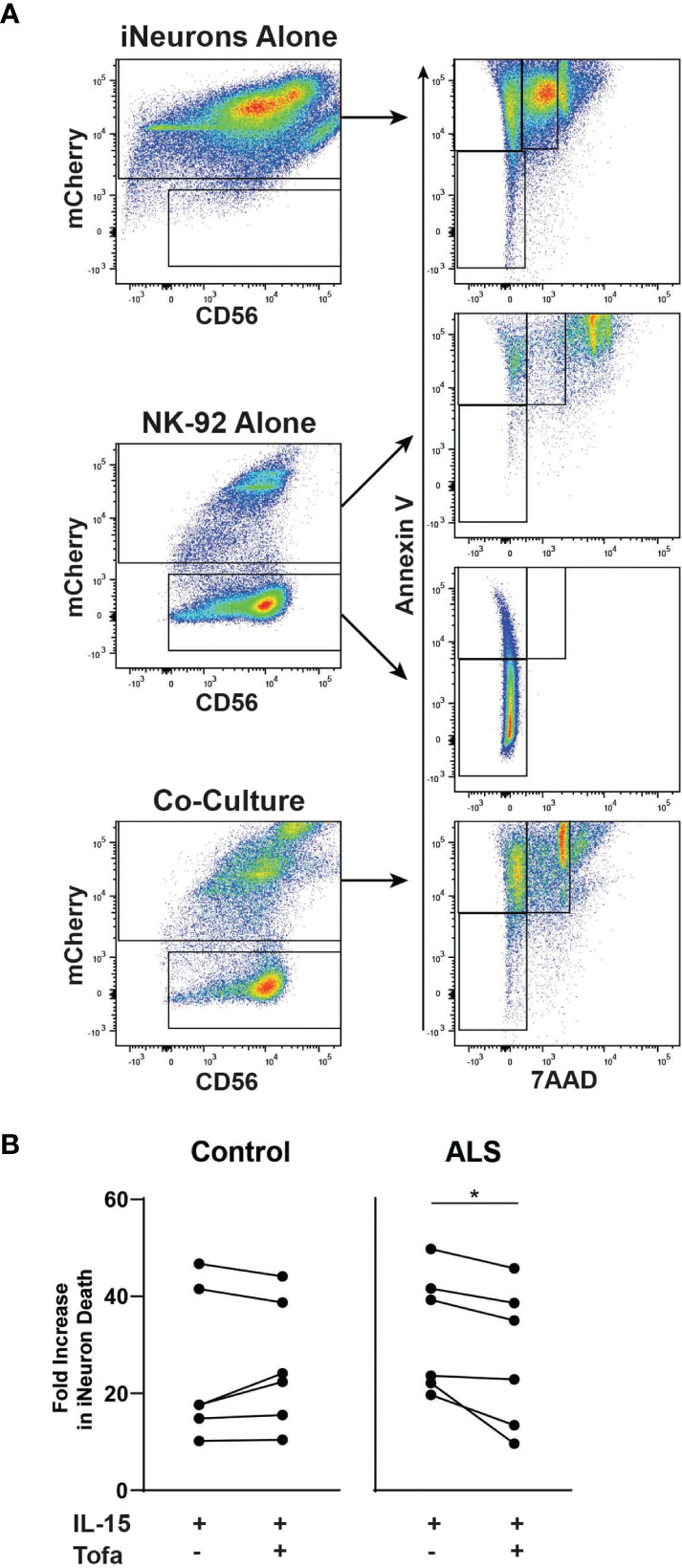
Tofacitinib decreases the cytotoxicity of NK-92 to motor neurons in an *in vitro* ALS model. iNeurons were differentiated from control- or ALS-participant derived iPSCs and were co-cultured for four hours with pretreated NK-92 cells (IL-15 ± tofacitinib); cell death was quantitated by flow cytometry. **(A)** Gating strategy for quantitating cell death of iNeurons cultured alone, of NK-92 cells cultured alone, and iNeuron and NK-92 cell co-culture. Dead iNeurons were characterized by positive fluorescence levels of annexin V (apoptosis) and moderate levels of 7AAD viability dye (cell death). **(B)** The rate of iNeuron death was quantitated in control- and ALS-derived iNeurons following co-culture with NK-92 cells; data were normalized to cell death rates from co-culture with Unstimulated NK-92 cells; n = 6 independent experiments. Comparisons were by paired t-test to assessed paired, non-normally distributed data. *P < 0.05.

Next, we explored whether tofacitinib protects neurons from NK cell-mediated cytotoxicity in an *in vitro* ALS model. To do so, we co-cultured NK-92 cells with iPSC-derived iNeurons. Two iNeuron cell lines were used: one derived from a control participant and one derived from an ALS participant (50). Following differentiation and ten days of growth, iNeurons were co-cultured for four hours with IL-15-stimulated NK-92 cells with and without tofacitinib. Annexin V and 7AAD were used as markers to quantitate iNeuron cell death by flow cytometry (43, 44), and mCherry was used to identify iNeurons within the co-culture (**Figure 2A**). However, dead NK-92 cells autofluoresce and appear positive for mCherry, and NK-92 cells cultured in iNeuron media #3 display increased NK cell death. Thus, dead NK-92 cells also appeared within the Annexin V+ and 7AAD+ gates for iNeurons, potentially skewing the data. To account for this this, we compared Annexin V and 7AAD flow plots for mCherry+ iNeurons versus mCherry+ NK-92 cells cultured alone in iNeuron media and found that NK 7AAD fluorescence levels for NK cells were higher than that of iNeurons. Thus, dead iNeurons were identified based on moderate 7AAD staining; cell death rates for iNeurons co-cultured with Stimulated and Treated NK-92 cells were then examined. We found that control-derived iNeurons showed similar rates of cell death whether cultured with or without tofacitinib treatment (**Figure 2B**). In contrast, there was a significant reduction in the rate of cell death for ALS-derived iNeurons that were cultured with Treated NK cells versus Stimulated NK cells. Together these results demonstrate that tofacitinib can protect ALS neurons from NK cell-mediated cytotoxicity.

### Tofacitinib Inhibits Primary NK Cells Function *Ex Vivo*

**Figure 3:**
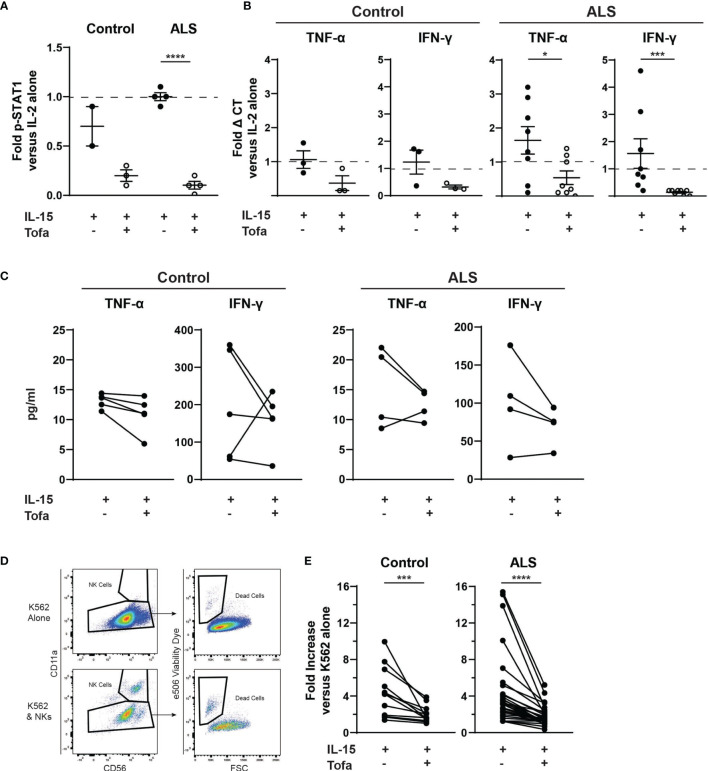
Tofacitinib inhibits primary NK cells *in vitro*. Primary NK cells were enriched from the whole blood of control and ALS participants. **(A)** P-STAT1 was assessed using Western blot. Primary NK cells were incubated with IL-2 alone, IL-2 + IL-15, or IL-2 + IL-15 + tofacitinib for 2 hours. Protein extracts were resolved by SDS-PAGE and immunoblotted for total STAT1, phosphorylated STAT1 (P-STAT1), and α-tubulin (internal reference). Graph represents densitometric analysis where total STAT1 and P-STAT1 signals were normalized to α-tubulin then normalized to NK cells receiving IL-2 alone; n = 2-4 participants. **(B)** Cytokine gene expression was assessed using qRT-PCR for *TNF-α* and *IFNγ*. Data were normalized to yWHAZ expression and normalized to the IL-2 NK cells; n = 3-8 participants. **(C)** Extracellular expression of TNF-α and IFN-γ was assessed for primary NK cells cultured with IL-15 ± tofacitinib (n = 5 control and n = 4 ALS). **(D)** Primary NK cells were assessed for cytotoxicity. Primary NK cells were cultured for two hours with K-562 cancer cells (1:1 ratio) + IL-15 ± tofacitinib; K-562 cell death was assessed using flow cytometry to quantitate e506 viability dye fluorescence. **(E)** Data were quantitated by normalizing to K-562 cells cultured without NK cells; n = 12 control and n = 32 ALS. For **(A, B)**, data are presented as mean ± SEM with dashed line showing cells cultures with IL-2 alone; comparisons were made by Student’s t-test. For **(C)**, comparisons were made using a paired t-test. For **(E)**, comparisons were made by Wilcoxon test to assessed paired, non-normally distributed data. *P < 0.05, ***P < 0.001, ****P < 0.0001.

We next extended these findings to determine whether tofacitinib suppresses cytotoxicity in primary NK cells from control and ALS participants (see **Table 1** for demographics). Primary NK cells were isolated from the peripheral blood of control and ALS participants. We first confirmed that tofacitinib suppresses JAK/STAT signaling in primary NK cells from control and ALS participants, similar to NK-92 cells. As measured by STAT1 phosphorylation *via* immunoblotting, tofacitinib significantly reduced P-STAT1 levels in primary ALS and control NK cells versus those stimulated with IL-2 and IL-15 (**Figure 3A**). Next, cytokine gene expression was examined in control and ALS primary NK cells following cytokine stimulation and treatment (**Figure 3B**). In ALS NK cells, tofacitinib treatment significantly decreased *TNF-α* and *IFN-γ* expression from primary ALS NK cells. A similar trend was observed in the cytokine gene expression of control primary NK cells. As with the NK-92 cell line, we also examined whether tofacitinib inhibits the release of pro-inflammatory and pro-apoptotic factors using a multiplex analysis. Conditioned media from primary NK cells cultured with IL-15 with and without tofacitinib was analyzed for the secretion of TNF-α and IFN-γ (**Figure 3C**). There was a trend towards reduced TNF-α secretion from primary NK cells isolated from control participants and reduced TNF-α and IFN-γ secreted from primary NK cells isolated from ALS participants.

**Table 1:** Subject demographics for primary NK cell analyses.

	Western Blot		Cytokine Gene Expression		Cytokine Protein Secretion		Cytotoxicity	
	Control (n = 3)	ALS (n = 4)	Control (n = 3)	ALS (n = 8)	Control (n = 5)	ALS (n = 4)	Control (n = 12)	ALS (n =32)
**Age(Mean ± SD) years**	70.5 ± 5.3	60.7 ± 13.4	66.2 ± 10.9	65.0 ± 8.2	69.32 ± 3.9	65.61 ± 6.7	61.7 ± 13.3	64.2 ± 9.0
**Sex (%) male**	66.6	25.0	33.3	37.5	60.0	50.0	50.0	56.3
**ALSFRS-R at Blood Draw**	N/A	23.0 ± 7.7	N/A	28.4 ± 10.3	N/A	35.8 ± 6.1	N/A	26.1 ± 8.3
**Site of Onset**	N/A	Bulbar (50.0%)Cervical (25.0%)Lumbar (25.0%)	N/A	Bulbar (25.0%)Cervical (25.0%)Lumbar (50.0%)	N/A	Bulbar (50.0%)Cervical (25.0%)Lumbar (25.0%)	N/A	Bulbar (18.8%)Cervical (34.4%)Lumbar (46.8%)
**Race**	White (100%)Black (0%)Asian (0%)Not reported (0%)	White (100%)Black (0%)Asian (0%)Not reported (0%)	White (100%)Black (0%)Asian (0%)Not reported (0%)	White (75.0%)Black (25.0%)Asian (0%)Not reported (0%)	White (100%)Black (0%)Asian (0%)Not reported (0%)	White (100%)Black (0%)Asian (0%)Not reported (0%)	White (100%)Black (0%)Asian (0%)Not reported (0%)	White (90.6%)Black (9.4%)Asian (0%)Not reported (0%)
**Ethnicity**	Not Hispanic (100%)Hispanic (0%)Not reported (0%)	Not Hispanic (100%)Hispanic (0%)Not reported (0%)	Not Hispanic (100%)Hispanic (0%)Not reported (0%)	Not Hispanic (100%)Hispanic (0%)Not reported (0%)	Not Hispanic (100%)Hispanic (0%)Not reported (0%)	Not Hispanic (100%)Hispanic (0%)Not reported (0%)	Not Hispanic (100%)Hispanic (0%)Not reported (0%)	Not Hispanic (100%)Hispanic (0%)Not reported (0%)
**Time from Onset to Collection (Mean ± SD) years**	N/A	5.0 ± 4.2	N/A	7.7 ± 6.8	N/A	2.7 ± 1.6	N/A	4.1 ± 3.9
**Time from Diagnosis to Collection (Mean ± SD) years**	N/A	3.6 ± 3.4	N/A	5.2 ± 6.7	N/A	1.6 ± 1.5	N/A	2.7 ± 3.6

N/A, not applicable.

Finally, we examined whether tofacitinib suppresses the cytotoxicity of primary NK cells isolated from control and ALS participants. After isolation, primary NK cells were co-cultured with K-562 target cancer cells, and the rate of K-562 cell death was used to quantitate primary NK cell cytotoxicity. Since K-562 viability can fluctuate, the rate of K-562 cell death in co-culture was normalized to the rate of K-562 cells cultured alone (**Figure 3D**). The cytotoxicity of both control and ALS primary Stimulated NK cells (IL-2 + IL-15) did not significantly differ from NK cells stimulated with IL-2 alone (data not shown). In contrast, primary NK cells treated with tofacitinib displayed significantly lower cytotoxicity to K-562 cells than Stimulated NK cells (**Figure 3E**). As with NK-92 cells, exposure to tofacitinib did not alter the viability of primary NK cells (data not shown). Together, these results demonstrate that primary NK cells are already stimulated in the peripheral blood of control and ALS participants, but tofacitinib can nevertheless suppress primary NK cell cytotoxicity by inhibiting JAK/STAT signaling.

### Tofacitinib Pharmacokinetics in Mice

Next, we wanted to optimize tofacitinib pharmacokinetics to administer to ALS mice in future studies, since NK cells are implicated in ALS pathogenesis (7, 13). However, symptoms in ALS mice do not emerge until after 90 days of age, and their lifespan is about 160 days of age in low-copy mouse strains (9). Therefore, preclinical studies of tofacitinib in SOD1^G93A^ mice will require long-term treatment. Unfortunately, previous studies testing tofacitinib in mice were either short-term studies with daily gavage (35) or longer-term studies using osmotic minipumps (51). For long-term diseases, administering tofacitinib daily by gavage is not logistically feasible and can induce significant animal losses (52). Conversely, minipumps cannot administer high treatment doses and require repeated surgeries. Thus, we wished to evaluate the efficacy of tofacitinib formulated into chow as a method of long-term administration for future preclinical studies.

Although tofacitinib pharmacokinetics had been performed in rats and human participants, little data are available in mice. Therefore, our first step was to assess tofacitinib pharmacokinetics after a single orally (PO, *per os*, gavage) or intravenously (IV) administered tofacitinib dose to male and female C57BL/6 mice. Blood was collected at multiple time points (5, 10, 15, 30, 60, 120, 240, 420, 960, 1440 minutes) post IV and PO administration and the kinetics of blood tofacitinib assessed. Tofacitinib bioavailability was roughly 37% in both male and female mice, *i.e*., roughly 37% of the initial dose administered orally reaches the peripheral blood (**Table 2**). Interestingly, we observed that other pharmacokinetic parameters differed between male and female mice. For instance, the maximal plasma level was higher in female versus male mice after both IV and PO dosing, while drug clearance was higher in male mice. These data indicate that tofacitinib can be administered orally by chow with similar drug uptake in males and females, though there may be sex-specific differences in drug metabolism resulting in altered plasma levels.

**Table 2:** Tofacitinib pharmacokinetic parameters in plasma following IV and PO administration.

Route	Sex	Dose	C0/Cmax	Tmax	AUC_(0-24)_	AUC_(0-inf)_	t½	CL/CL_F	Vss/Vz_F	%F
Unit		mg/kg	ng/mL	h	h*ng/mL	h*ng/mL	h	mL/h/kg	mL/kg	%
**IV**	M	10	3554.4	N/A	676.76	678.09	1.85	14747.23	39278.78	N/A
**PO**	M	20	960	0.25	505.74	508.48	0.91	39332.99	51817.31	37.4
**IV**	F	10	6516.9	N/A	980.54	982.90	0.72	10173.94	10549.83	NA
**PO**	F	20	1114.3	0.167	713.26	719.57	0.90	27794.47	36093.12	36.4

IV, intravenous, PO, per os; C0, concentration at time 0; Cmax, maximum observed concentration; Tmax, time to reach Cmax; AUC_(0-24)_, area under the concentration-time curve from time zero to 24 hours; AUC_(0-inf)_, area under the concentration-time curve from time zero to infinite; CL, systemic clearance; CL_F, apparent clearance; Vss, volume of distribution at steady state; Vz_F, volume of distribution associated with the terminal elimination phrase; terminal elimination half-life (t½) was calculated based on data points (≥3) in the terminal phase with correlation of coefficient >0.90; %F, bioavailability; N/A, not applicable.

### Efficacy of Low- and High-Dose Tofacitinib in Mouse Chow

**Figure 4:**
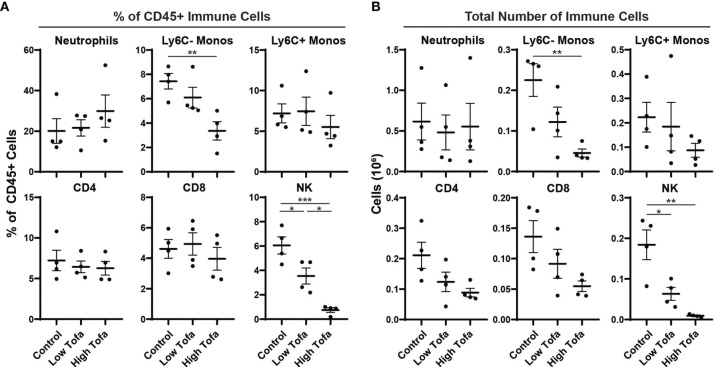
Impact of orally administered tofacitinib on peripheral immune populations in mice. WT control mice (half male and half female) were treated for two weeks with low- (5 mg/kg) and high- (30 mg/kg) dose tofacitinib administered in standard chow. Immune cells were then analyzed in peripheral blood using flow cytometry; **(A)** percentage of all CD45+ immune cells as well as **(B)** total numbers of cells was examined for six immune populations. Data show mean ± SEM. Comparisons by ANOVA; n = 4 mice per group. *P<0.05, **P<0.01, ***P<0.001; monos, monocytes.

Finally, we tested tofacitinib efficacy formulated in chow on NK cell levels in mice. Based on the pharmacokinetic data, chow was formulated to deliver a daily dose of 5 mg/kg (low-dose) or 30 mg/kg (high-dose) to male and female WT control mice (*i.e*., WT littermates on an SOD1^G93A^ background) for two weeks. At the end of the treatment period peripheral immune cell levels were assessed by flow cytometry for the percentage and total number of NK cells, neutrophils, Ly6C- monocytes, Ly6c+ monocytes, CD4 T cells, and CD8 T cells. Both low- or high-dose tofacitinib treatment significantly lowered NK cell percentage in a dose-dependent manner (**Figure 4A**). Moreover, both doses significantly reduced total NK cell counts in peripheral blood versus normal chow, and there was a trend towards fewer circulating NK cells in of high- versus low-dose mice (**Figure 4B**). In contrast, tofacitinib treatment did not significantly reduce the percentage or total number of neutrophils, Ly6C+ monocytes, CD4 T cells, or CD8 T cells. Interestingly, high-dose mice had significantly lower percentage and total number of circulating Ly6C- monocytes, which is consistent with our previous study utilizing NK cell depletion (13). Together, these results demonstrate that tofacitinib can be administered in chow mouse models and suppresses NK cell levels in a dose-dependent manner.

## Discussion

Previous studies have shown that tofacitinib treatment suppresses NK cell levels (34–36, 46), but there is limited information on the impact of tofacitinib on NK cell function (41). Similarly, preclinical mouse disease studies of tofacitinib have been short-term or used administration routes unsuitable to long-term studies. Addressing these shortcomings is of utmost importance to test tofacitinib for regulating NK cell function and counts in future preclinical ALS studies, since NK cells are implicated in ALS progression (7, 13). Therefore, in the current study, we evaluated tofacitinib on NK cell function *in vitro* and conducted a pharmacokinetic study *in vivo*. We used the pharmacokinetic data to formulate tofacitinib in chow, a suitable format for long-term oral administration. We evaluated the impact of a 2-week tofacitinib chow regimen on circulating NK cell levels in WT mice. We found tofacitinib suppressed IL-15-mediated JAK/STAT pathway stimulation, cytotoxicity to cancer cells and iNeurons, granzyme B and perforin expression, and cytokine expression in the NK-92 cell line. Importantly, in the context of ALS, tofacitinib also significantly lowered cytotoxicity of IL-2/IL-15-stimulated primary NK cells isolated from ALS participants and healthy controls, as well as cytokine levels. Finally, tofacitinib bioavailability was similar in male and female mice, although there were sex differences in some parameters; formulation in chow at both low- (5 mg/kg) and high-dose (30 mg/kg) tofacitinib after 2 weeks lowered peripheral NK cell levels in WT control mice.

These findings suggest that tofacitinib may be a viable therapeutic strategy to regulate the NK cell population in ALS. NK cells accumulate in the spinal cord of ALS mice (8, 13, 19, 53). In individuals with ALS, NK cells are increased in the peripheral blood (10, 11) and co-localize with motor neurons in *postmortem* spinal cord tissue, driving microglial activation *via* IFN-γ expression (7). Indeed, as ALS progresses, motor neurons lose surface markers, which protect against NK cells-mediated cytotoxicity (20), rendering them more susceptible to attack. In addition to increased NK cell levels, they are also more highly activated in individuals with ALS, correlating with disease progression (13). Depleting NK cells from SOD1^G93A^ ALS mice extends survival (7, 13). Thus, reducing NK cell levels, blocking NK cell cytotoxicity, and suppressing IFN-γ release from NK cells may slow motor neuron loss and suppress central nervous system inflammation, increasing survival in ALS.

Previous studies have established that tofacitinib blocks immune cell activation and activity by interfering with the JAK/STAT pathway (25), promoting pro-inflammatory cytokine signaling between cells (27, 54, 55). Blocking cytokines, such as IFN-γ, with tofacitinib prevents inflammatory T cell activation, which effectively treats autoimmune disorders, such as rheumatoid arthritis, ulcerative colitis, and psoriasis (22–24). In the case of NK cells, blocking JAK/STAT signaling suppresses the IL-15 pathway (33), which is a crucial mediator of both NK cell survival and activation (29, 56, 57). This likely explains the reduced peripheral NK cell levels observed after tofacitinib treatment in both mouse and humans (34–36, 46). However, in addition to maintaining NK cell homeostasis, IL-15 is also a potent stimulator of NK cell function (30–32). Thus, tofacitinib should also block IL-15-mediated NK cell activation. Our current study definitively shows this: tofacitinib treatment suppresses pro-inflammatory cytokine production and cytotoxicity in both NK-92 cells and primary NK cells. This is particularly salient to ALS, since direct NK cell cytotoxicity as well as IFN-γ production likely contribute to disease progression (7). The importance of these findings is further corroborated by our results showing that tofacitinib significantly reduces NK cell cytotoxicity towards iNeurons generated from ALS patient-derived iPSCs.

The current study also suggests that tofacitinib suppresses NK cell levels in an ALS mouse model. However, although preclinical mouse models are crucial for evaluating drug efficacy *in vivo*, no comprehensive studies have examined tofacitinib pharmacokinetics nor the long-term impact of the drug on peripheral immunity in mice. There are challenges associated with long-term drug administration. One possible solution is the use of osmotic minipumps as it has been previously shown (51). However, minipumps typically administer either a low drug dose over a long period of time or a high dose over a short period of time. A higher dose of tofacitinib, such as 30 mg/kg, would require frequent pump replacement and multiple surgeries. Not only is this logistically difficult, but frequent surgeries would be potentially life-threatening for mice in advanced stages of disease. In contrast, daily oral tofacitinib administration tofacitinib to mice *via* gavage is not logistically feasible over long time periods either as the rate of death associated with technique is 15% over a six week period (35, 52). Moreover, these previous studies did not examine tofacitinib pharmacokinetics – particularly bioavailability – meaning the final concentration in the peripheral blood following oral administration was not known. In the present study we found that tofacitinib bioavailability in mice (around 37%) differed from humans, where bioavailability is 74% (58). Perhaps unsurprisingly, bioavailability in mice is closer to that in rats (29%) (59). Interestingly, we found that plasma tofacitinib levels differed between male and female mice, even after IV administration, suggesting the sexes may clear the drug at different rates. These sex-specific tofacitinib pharmacokinetics differences are potentially important for future ALS treatment, since we have previously described sex-based immune differences in ALS (12, 13).

Consistent with other methods of tofacitinib administration, treating mice orally with tofacitinib in chow successfully suppressed circulating NK cell levels in a dose-dependent manner in WT mice on a SOD1^G93A^ genetic background. Together with our *in vitro* findings, these results suggest that tofacitinib modulates NK cell levels and activity and should be tested in preclinical mouse models of ALS. However, an in-depth series of studies will be required, as the mechanisms of NK cell involvement in ALS is incompletely understood. One mechanism by which NK cells contribute to ALS is the destruction of damaged motor neurons within the CNS, as motor neurons are uniquely vulnerable to NK cells during disease progression (20). Alternatively, NK cells may be involved in other disease mechanisms. NK cells may play an important role in driving early microglial activation (7) which has been implicated in ALS pathology (60–62), and they may also contribute to peripheral nerve damage in ALS, as increased expression of major histocompatibility complex I was associated with slower disease progression in mouse models of ALS (63). The role of NK cells in the loss of neuromuscular junction (NMJ) integrity during ALS has also not been examined.

Preclinical studies must also account for the impact of tofacitinib on other immune cell populations that modulate ALS progression, both in the periphery and the CNS. Immune cells are both protective and destructive in ALS (2, 10), so preserving protective immune function is of the utmost importance when designing and utilizing immune-based therapies. While the current study demonstrated that tofacitinib suppresses NK cell numbers in the peripheral blood, changes were also observed in other cell populations. Ly6C- monocytes, which patrol the body and are involved in fibrosis and wound repair (64), were significantly reduced in mice treated with the higher tofacitinib dose; analogous monocytes in human patients may have a protective effect (65). Similarly, there was a trend towards reduced CD4 and CD8 T cell levels following tofacitinib treatment, particularly in mice treated with the high dose. While these observations did not reach statistical significance, it is important to account for these changes, as these immune cell types play a central role in ALS, in particularly CD4 T cells (4, 10). Since the cellular lifespan of NK cells (66–68) is much shorter than that of T cells (69) it may be possible to preserve T cell levels by utilizing on/off drug treatment cycles. This possibility should also be explored in future clinical trials.

In addition, preclinical tofacitinib studies should account for the impact of sex in ALS mouse models. Though the impact of sex on tofacitinib-NK cell interaction was not explored in the current study, we have previously demonstrated that sex alters the impact of several immune cell populations in ALS, including NK cells (12, 13). Moreover, depletion of NK cells also impacts male and female mice differently, both in terms of survival and neuroinflammation (13); therefore, tofacitinib studies should likewise account for sex differences given the reduction in peripheral NK cell levels following tofacitinib treatment. Altogether, *in vivo* tofacitinib studies in ALS mice should examine a myriad of mechanisms and factors including peripheral and CNS immune cell populations, peripheral and CNS gene expression, motor neuron survival, and NMJ integrity. Studies will need to examine drug dosing, drug timing, and will need to account for the impact of sex.

The current study does have several limitations. First, primary NK cells are more difficult to culture than NK-92 cells and were therefore not co-cultured with iNeurons. iNeurons are also not motor neurons, thus, it is unclear to what degree ALS iNeurons recapitulate true motor neurons *in vivo*. Moreover, in mice, we only examined the effect of tofacitinib on NK cell numbers rather than on NK cell function. Many of the *in vitro* assays require large cell numbers, and tofacitinib treatment reduced overall NK cell levels, making these analyses *in vivo* difficult. Finally, while we have previously shown that both age and sex alter the activity of immune cells during ALS, including NK cells (12, 13), the present study did not explore the impact of these factors on tofacitinib suppression of NK cells. Nonetheless, our results conclusively show that tofacitinib suppresses NK cell function *in vitro*, suppresses NK cell levels *in vivo*, and can be administered orally in chow for use in preclinical ALS mouse models. These findings also indicate tofacitinib may be used to treat long-term diseases mediated by NK cell function, such as ALS.

## Data Availability Statement

The raw data supporting the conclusions of this article will be made available by the authors, without undue reservation.

## Ethics Statement

The studies involving human participants were reviewed and approved by University of Michigan Medical School Institutional Review Board. The patients/participants provided their written informed consent to participate in this study. The animal study was reviewed and approved by University of Michigan Institutional Animal Care & Use Committee.

## Author Contributions

CF-R, AM, BM, and EF designed the overall study. CF-R, AM, BM, JF, IW-D, CEP, ST, and CP performed *in vitro* studies using NK-92 cells. CF-R, BM, IW-D, ST, and CP performed iNeuron co-culture assays. CF-R, BM, JF, IW-D, CEP, ST, and CP performed *ex vivo* studies using primary NK cells. BM, SG, and EF wrote the IRB protocol. SG and EF recruited human participants for the study. JF and CEP worked with the pharmacokinetic core for the mouse pharmacokinetic studies. BM, JF, and CEP performed the mouse immune studies. CF-R, AM, and BM analyzed the data. CF-R, AM, BM, JF, IW-D, CEP, ST, CP, SG, and EF wrote the manuscript with substantial input from all authors. All authors contributed to the article and approved the submitted version.

## Funding

This work was partially supported by the U.S. National Institutes of Health (R21NS102960 and R01ES030049 to EF, K23ES027221 to SG, and T32NS0007222 supported AMM-C), the Centers for Disease Control and Prevention/Agency for Toxic Substances and Disease Registry (CDC/ATSDR; R01TS000289 to EF), the ALS Association (20-IIA-431 to BM), and the Department of Defense (W81XWH-21-1-0293 to BM). Support was also provided by the Sinai Medical Foundation Neuroscience Scholar Fund and the NeuroNetwork for Emerging Therapies at the University of Michigan.

## Conflict of Interest

BM, SG, and EF are listed as inventors on a patent, Issue number US10660895, held by University of Michigan titled “Methods for Treating Amyotrophic Lateral Sclerosis” that targets immune pathways for use in ALS therapeutics.

The remaining authors declare that the research was conducted in the absence of any commercial or financial relationships that could be construed as a potential conflict of interest.

## Publisher’s Note

All claims expressed in this article are solely those of the authors and do not necessarily represent those of their affiliated organizations, or those of the publisher, the editors and the reviewers. Any product that may be evaluated in this article, or claim that may be made by its manufacturer, is not guaranteed or endorsed by the publisher.
